# Xuebijing Protects Rats from Sepsis Challenged with *Acinetobacter baumannii* by Promoting Annexin A1 Expression and Inhibiting Proinflammatory Cytokines Secretion

**DOI:** 10.1155/2013/804940

**Published:** 2013-11-28

**Authors:** Xian-Di He, Yan Wang, Qiong Wu, Hua-Xue Wang, Zhen-Dong Chen, Rong-Sheng Zheng, Zi-Shu Wang, Jun-Bin Wang, Yan Yang

**Affiliations:** ^1^Department of Intensive Care, The First Affiliated Hospital of Bengbu Medical College, Bengbu 233004, China; ^2^Department of Nursing, The First Affiliated Hospital of Bengbu Medical College, Bengbu 233004, China; ^3^Department of Medical Oncology, The First Affiliated Hospital of Bengbu Medical College, Bengbu, Anhui 233004, China

## Abstract

Xuebijing (XBJ) injection is a herbal medicine that has been widely used in the treatment of sepsis in China; however, its role in the development and progression of *Acinetobacter baumannii* sepsis and the underlying mechanisms remain uninvestigated. In the present study, fifty-four male Wistar rats were randomly assigned to normal-control group, sepsis-control group, and sepsis + XBJ group, each containing three subgroups of different treatment time periods (6, 12, and 24 hrs following injection, resp.). The sepsis model was established by intraperitoneal injection of *A. baumannii* ATCC 19606. For XBJ treatment, 4 mL/kg XBJ was administrated simultaneously by intravenous injection through caudal vein every 12 hrs. All animals demonstrated ill state, obvious intestinal dysfunction, histopathological lung damages, and overactive inflammatory responses after *A. baumannii* infection, and these events could be partially reversed by XBJ treatment from the beginning of infection. XBJ induced an increase in the expression of anti-inflammatory mediator annexin A1; however, two proinflammatory cytokines, interleukin-8 (IL-8) and tumor necrosis factor-**α** (TNF-**α**), were decreased at the each monitored time point. These findings suggested that XBJ via its cytokine-mediated anti-inflammatory effects might have a potential role in preventing the progression of *A. baumannii* infection to sepsis by early administration.

## 1. Introduction

*Acinetobacter baumannii* (*A. baumannii*) is a Gram-negative coccobacillus associated primarily with nosocomial infections. This microorganism has relevant clinical implications as it survives on almost every surface and develops resistance to all available antibiotics [1]. Evidence suggests that *A. baumannii* infections can be shown to manifest as bacteremia, pneumonias, meningitis, urinary tract infections, surgical site infections, and even sepsis [2, 3]. Sepsis is a systemic inflammatory response to infection that is initiated by bacteria and their related toxins. Ranging from the systemic inflammatory response syndrome (SIRS) and its complications septic shock and multiple organ dysfunction syndrome (MODS), sepsis represents the leading cause of death in intensive care patients. Despite the use of combination antibiotic application and good supportive therapy and care, treatment for sepsis is still unsatisfactory, and mortality from severe sepsis remains high between 34 and 43% [4]. Therefore, urgent needs exist for both advances in the understanding of sepsis pathogenesis and new agents to treat *A. baumannii *infection.

Inflammation plays key roles in sepsis [5]. A most prominent pathological feature of sepsis has been demonstrated to be associated with an exuberant, uncontrolled inflammatory response to microbial products [6]. Generally, when a host is exposed to bacterial toxins, immune cells are activated, and various proinflammatory cytokines are over-secreted, which can result in imbalanced metabolic pathways and tissue damage. To prevent severe damage, anti-inflammatory mediators are released. With advances in the understanding of the proinflammatory versus anti-inflammatory immune responses during sepsis in recent years, studies have demonstrated that both types of cytokines are regulated simultaneously at the onset of sepsis [7, 8] and that either cytokine was reliable in predicting mortality [9, 10]. Proinflammatory cytokines, such as tumor necrosis factor-*α* (TNF-*α*), interleukin-1 (IL-1), IL-6, and IL-8, are necessary to initiate an effective inflammatory process in patients with sepsis [11]; however, somewhat to the contrary, anti-inflammatory cytokines such as IL-10, IL-13, IL-4, and transforming growth factor-*β* (TGF-*β*), are also found to be in a relative high level in septic patients [12, 13]. These findings implicate that drugs multitargeting inflammatory mediators will provide a promising strategy in treatment of sepsis or even protect hosts from sepsis.

In the recent septic immunomodulatory study, the traditional Chinese medicine (TCM) attracts much attention for its therapeutic concepts of integrated and balanced regulation. Xuebijing (XBJ) is a newly developed injection concocted from extracts of five Chinese herbs (i.e., *Carthamus tinctorius* or safflower, *Paeoniae radix* or red peony root, *Salvia divinorum* or Diviner's Sage, *Angelica sinensis* or “female ginseng”, and *Ligusticum wallichii Franchet* or *Chuanxiong* in Chinese). It shows satisfactory antiendotoxin and anti-inflammatory effects in a series of animal experiments [14–17] and was approved by the positive results of clinical trials for the treatment of certain disease such as sepsis or MODS in China. However, prior studies focused more on the immuno-modulatory activity of XBJ after the onset of sepsis, little is known about its effect of early prophylactic administration on the development and progression of sepsis especially challenged with *A. baumannii*. That is, when XBJ is given simultaneously the first moments of *A. baumannii *infection, whether it may retard or alleviate sepsis-related tissue injury and overactive inflammatory reaction remains to be elucidated. Thus, the aim of the present study was to evaluate the *in vivo* activity of XBJ in the development of *A. baumannii* sepsis in an experimental rat model and its dynamic effects on proinflammatory cytokines TNF-*α* and IL-8 and anti-inflammatory mediator annexin A1.

## 2. Materials and Methods

### 2.1. Chemicals and Reagents

XBJ was supplied by Tianjin Chase Sun Pharmaceutical Co., Ltd. (Tianjin, China). Dextran and 3,3-diaminobenzidine tetrahydrochloride (DAB) were purchased from Sigma-Aldrich (St. Louis, MO, USA). The antibodies for annexin A1 and *β*-actin were purchased from Santa Cruz Biotechnology (USA). Secondary antibodies for western blotting and immunohistochemistry were from Amersham Biosciences Corp. (Piscataway, NJ, USA). Enzyme-linked immunosorbent assay (ELISA) was performed via a commercially available kit (R&D systems, Minneapolis, MN, USA). All other chemicals used were of analytical reagent grade.

### 2.2. Bacterial Strains

The commercially available quality control strain of *A. baumannii* ATCC 19606 was used as previously described [18]. This organism was isolated from a clinical specimen and was characterized by routine assays: it results in resistance to all commonly used antibiotics, with the exception of colistin and tigecycline. The isolate was stored at −80°C until required.

### 2.3. Animals

A total of 54 adult male Wistar rats weighing 250 to 300 g were used for all the experiments. They were housed in individual cages under constant temperature (22 ± 2°C) and humidity with 12 hrs light/dark cycle and had access to chow and water *ad libitum *throughout the study. All experimental procedures were approved by the local institutional review board (Bengbu Medical College, Bengbu, China (authorization no. 2012–018, Mar 19, 2012)) and performed according to the regulations of the National Institutes of Health Guide for the Use of Laboratory Animals.

### 2.4. Sepsis Model

An experimental sepsis model was established by *A. baumannii *injection, as previously described with some modifications [18]. Briefly, *A. baumannii *ATCC 19606 was grown in nutrient broth. When bacteria were in the log phase of growth, the suspensions were centrifuged at 4000 g for 5 minutes, the supernatants were discarded, and the bacteria were resuspended and diluted into sterile saline. Rats were anesthetized with an intraperitoneal injection of pentobarbital sodium (20 mg/kg of body weight). The abdomen of each animal was shaved and prepared with iodine. The animals then received intraperitoneally 10 mL/kg saline containing 7 × 10^8^ colony forming units (CFU)/mL of *A. baumannii *ATCC 19606, at which concentration a lethality rate of about 50% was observed within 48 hrs after injection as shown in our previous study [19].

### 2.5. Study Groups

**Figure 1:**
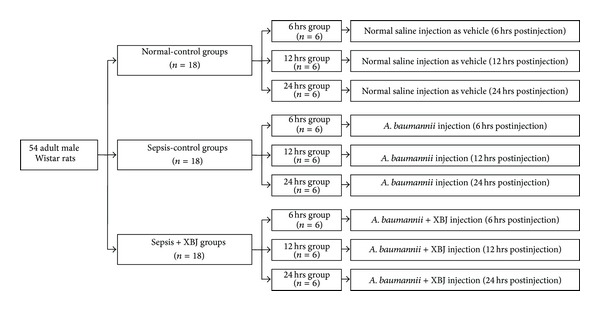
Animal grouping. 54 adult male Wistar rats were used for experiments and allocated to the different time points as indicated. Normal-control groups (*n* = 18) were administrated with normal saline both intraperitoneally (10 mL/kg) and caudal intravenously (4 mL/kg); sepsis-control groups (*n* = 18) were concurrently administrated with *A. baumannii *ATCC 19606 (10 mL/kg saline containing 7 × 10^8^ CFU/mL) intraperitoneally and normal saline (4 mL/kg) caudal intravenously; sepsis + XBJ groups (*n* = 18) were concurrently administrated with *A. baumannii *ATCC 19606 (10 mL/kg saline containing 7 × 10^8^ CFU/mL) intraperitoneally and XBJ injection (4 mL/kg) caudal intravenously. All rats from each group was survived at each monitored time point.

54 rats were randomly assigned to three normal-control groups (with normal saline injection both intraperitoneally (10 mL/kg) and caudal intravenously (4 mL/kg)), three sepsis-control groups (with *A. baumannii* (10 mL/kg) injection intraperitoneally and normal saline (4 mL/kg) injection caudal intravenously), and three sepsis + XBJ groups (with *A. baumannii* (10 mL/kg) injection intraperitoneally and XBJ injection caudal intravenously). XBJ (4 mL/kg) was given every 12 hrs by intravenous injection from tail vein. According to prior study, after being subjected to *Acinetobacter *strains, the organisms disseminated from the peritoneum to distant organs by 4 hrs after inoculation [20], and all animals would be bacteraemic within 24 hrs [21]. Thus, we defined the time points as 6, 12, and 24 hrs after injection, respectively, to observe the dynamic protective effect of XBJ during the development of *A. baumannii *sepsis. The use of this paradigm also allows us to compare the therapeutic efficacy between “short” and “long” administration of XBJ. The specific intervention established in each group is detailed in Figure 1.

### 2.6. Histopathological Examination

Ten specimens in each group were examined. All specimens were sent for histopathological examination using the classical hematoxylin and eosin (H&E) staining technique after fixation in formalin. They were embedded in paraffin and cut by a microtome into 4 *μ*m sections that were stained by routine H&E. The specimens were then examined microscopically.

### 2.7. Isolation of Blood Neutrophils

Neutrophil isolation was determined by a “Ficoll-Hypaque gradient centrifugation and red blood cell lysis” technique as previously described [22, 23]. Briefly, blood (8–10 mL) was collected from carotid artery into heparinized syringes. Heparinized whole blood diluted 1 : 1 with PBS was then layered onto Ficoll-Hypaque (Pharmacia, Peapack, NJ, USA; specific gravity 1.077) and centrifuged (400 g, 30 min). The erythrocyte/granulocyte pellet was diluted 1 : 1 with normal saline. Erythrocytes were sedimented by 3% dextran in 0.9% saline and incubated for 1 hr. Supernatants were collected and centrifuged for 10 min at 4°C. The remaining red blood cells were lysed in a lysing solution of NH_4_Cl. The neutrophils were then washed and resuspended in D-Hanks balanced salt solution (HBSS). Differential counts using crystal violet staining of the nuclei revealed that ≥95% of the cells were neutrophils.

### 2.8. Western Blotting for Annexin A1 in Neutrophils

Protocols for western blotting are described in our previous studies [24, 25]. Using antibodies at dilution recommended by the suppliers: mouse anti-annexin A1 IgG as primary antibody at 1 : 400 dilution and alkaline phosphatase-conjugated goat anti-mouse IgG as secondary antibody at 1 : 10000 dilution. The immunoreactive bands were visualized by Amersham ECL Plus Western Blotting Detection Kit (GE Healthcare, Piscataway, NJ, USA). All western blotting exposures were in the linear range of detection, and the intensities of the resulting bands were quantified by Quantity One software with a GS-800 densitometer (Bio-Rad).

### 2.9. Immunohistochemical Analysis for Annexin A1 in Lungs

Samples in each group were prepared as described above. All sections were deparaffinnized in xylene and dehydrated using graded series of alcohol. The endogenous peroxidase activity was quenched by incubation in methanol containing 3% H_2_O_2_ for 10 min at room temperature then heated for 30 min at 95°C to repair antigens and finally rinsed in phosphate-buffered saline (PBS). The sections were blocked using 3% BSA in PBS for 1 hr at room temperature, and then incubated overnight at 4°C with specific primary antibody for annexin A1 (1 : 100 dilution). After several washes in PBS, the slides were incubated with secondary antibody IgG-HRP (1 : 400 dilution) in PBS and 0.05% Tween 20 at room temperature. After a complete wash in PBS, the slides were developed in freshly prepared DAB solution for 8 min and then counterstained with hematoxylin followed by dehydration and mounted. A negative control was included in each immunostaining, in which the first antibody was replaced by 3% BSA. Quantitative analysis was made in a blinded manner under a light microscope. The evaluation of annexin A1 staining was done according to the scoring system described by Hongsrichan et al. [26], based on signal intensity and positive area as follows: 0, negative, <10%; 1, weak (+), 10%–25%; 2, moderate (++), 26%–75%; and 3, strong (+++), >75%. The final score was determined by multiplying the intensity of positivity and the extent of positivity scores.

### 2.10. Collection of Serum Samples and Cytokine Assay

2 mL blood samples of individual group were taken from the heparin lock of the carotid artery catheter 6, 12, and 24 hrs after the first injection. The blood samples were placed in 1.5 mL microtubes and centrifuged at 4000 rpm for 15 min to separate plasma. Serum samples were then removed and stored at −80°C until use. Cytokine level in rat serum was measured by the ELISA kit (R&D Systems) according to the manufacturer's instructions. The intensity of the color was measured using a microplate ELISA reader (MRX II, Dynex Technologies, Chantilly, VA, USA) by reading the absorbance at 450 nm. The results for the samples were compared with the standard curve to determine the concentrations of IL-8 and TNF-*α*.

### 2.11. Statistical Analysis

Statistical analyses were performed with SPSS software (Version 19.0). All data were expressed as mean ± S.D. Differences among groups were analyzed by one-way analysis of variance using the Duncan test. Differences with *P* < 0.05 were considered significant.

## 3. Results

### 3.1. Comparison of General Condition of the Rats

All animals were observed up to 24 hrs after inoculation and no rat died prior to the designated time point for analysis. Timing of investigation was set at 6 hrs, 12 hrs, and 24 hrs after injection, respectively, allowing us to confirm the development and progression of sepsis and also observe the role of XBJ in intervening in this sequence of events. We found that control rats experienced a good condition and state; however, 6 to 12 hrs after *A. baumannii* injection, rats became lethargic and developed fever, piloerection, diarrhea, huddling, and malaise, all the symptoms of sepsis. As the observation period lengthened to 24 hrs, rats barely moved and exhibited dyspnea and a dramatic decrease in body temperature. Treatment with XBJ for the same time period improved the symptoms and signs, although a decrease in autonomic activities was observed, and the anal temperature was approximately 37°C (data not shown).

### 3.2. Gross Observation of Peritoneal Cavity

**Figure 2:**
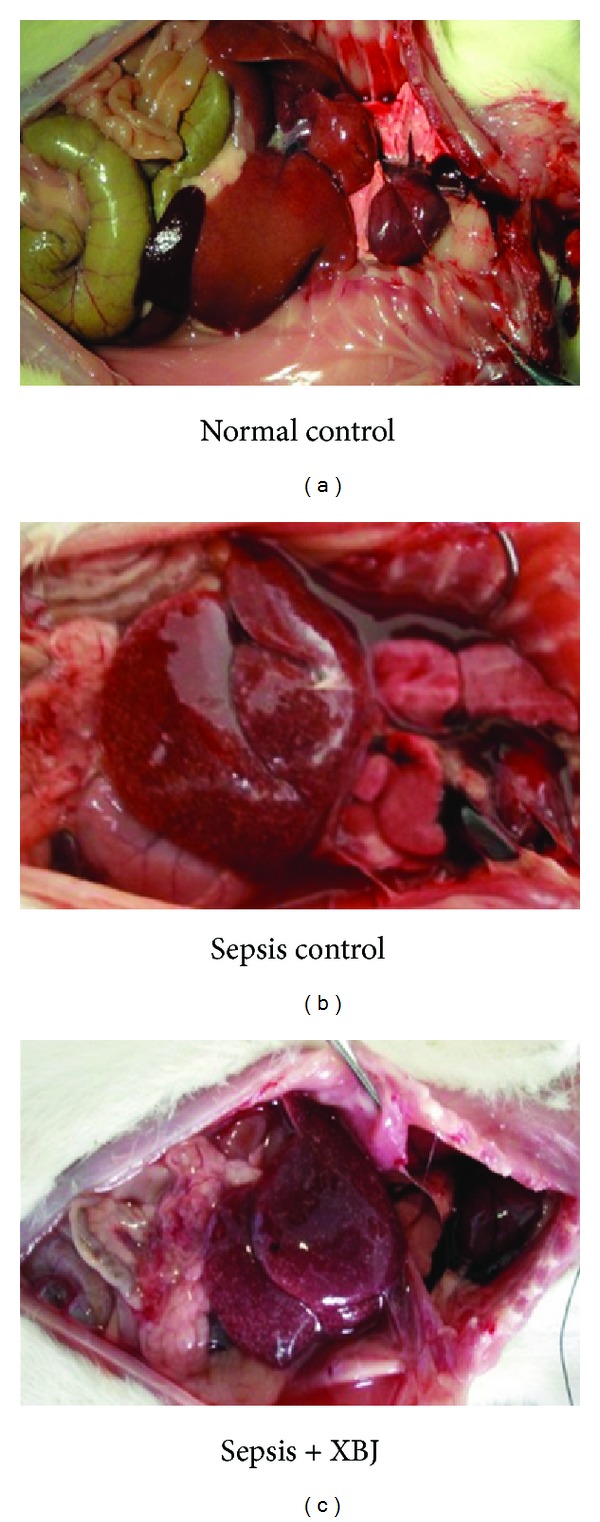
Macroscopic findings of peritoneal cavity. Rats were from normal-control, sepsis-control, and sepsis + XBJ groups at 12 hours after injection, after which the rats were sacrificed and gross observations of intraperitoneal injuries were conducted. Experiments were performed with all rats and representative photographs were shown.

At the time of sacrifice, the abdominal wall was exposed and the peritoneal cavity was then opened. Compared with saline controls, peritoneal effusion, serious intestinal flatulence, dilated but pale bowels, and intestinal hypoperistalsis or even the presence of ascitic fluid could be seen in rats of sepsis-control groups. And the cascade of these disturbed events became significantly more pronounced as the time prolonged. This feature of intestinal loading thus provided proof that the rats in the sepsis-control group experienced an active bacterial infection. Peritoneal effusion in sepsis + XBJ group was detected but less than that of sepsis-control group, eliciting almost ruddy bowel and good peristalsis at either indicated time point. The representative images of three treatment groups by 12 hrs after inoculation were shown in Figure 2.

### 3.3. Histopathological Examination of Lungs

**Figure 3:**
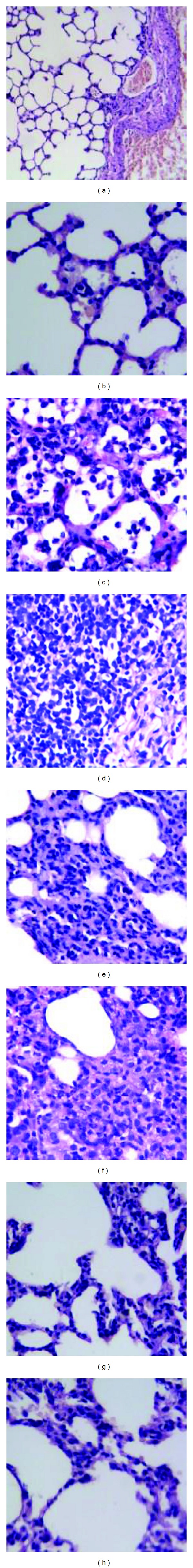
Histopathological studies by light microscope showing morphologically lung tissues from rats in the (a) and (b) normal-control, (c)–(e) sepsis-control, and (f)–(h) sepsis + XBJ groups at 6 (c) and (f), 12 (d) and (g), and 24 (e) and (h) hours after injection, respectively. An acute perivascular and alveolar inflammation was observed already after 6 hours following *A. baumannii* infection, and it became massive accompanied by diffuse damage resulting ultimately in the destruction of alveolar structures and thickening of the septal space at a late phase; however, simultaneous administration of XBJ persistently attenuated the above indicators leading to small changes in alveolar architecture and histology during the 24-hour observation period. Original magnification: (a) ×100 and (b)–(h) ×400.

The histopathologic findings of the lung tissues with H&E staining showed that the pulmonary architecture was normal in control group (Figures 3(a) and 3(b)). In contrast, an acute inflammation in the interstitium and alveolar spaces characterized by neutrophils and macrophages infiltrations was already observed in sepsis-control group at the early 6 hours after injection (Figure 3(c)). As the infection time prolonged, more infiltrating cells, including red blood cells, throughout the lung tissue were detected (Figure 3(d)), and ultimately the destruction of alveolar structures and thickening of the alveolar wall were shown at the late phase of infection (Figure 3(e)). However, lungs harvested in the sepsis + XBJ groups had less intraalveolar and interstitial patchy congestion and hemorrhage. Meanwhile, neutrophil infiltration was reduced, and the destruction of lung structure was significantly lightened, compared to that of sepsis-control groups for the same time period (Figures 3(f), 3(g), and 3(h)).

### 3.4. Annexin A1 Expression in the Process of Sepsis and Its Modulation by XBJ

**Figure 4:**
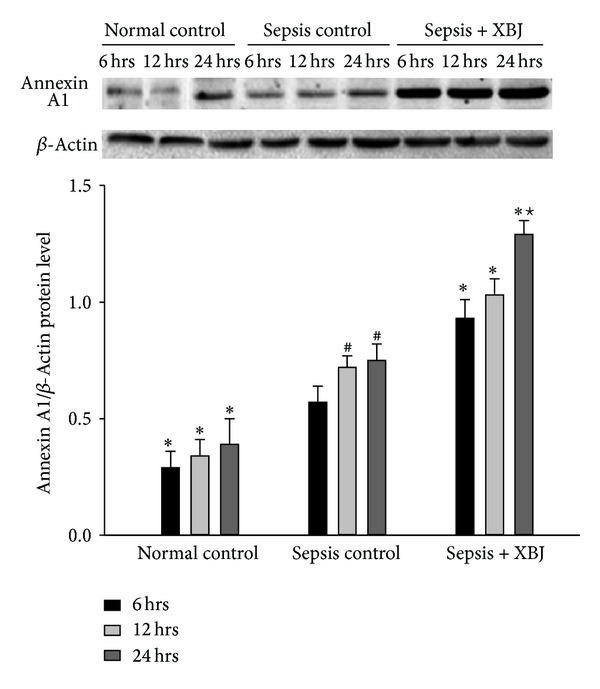
Expression level of annexin A1 protein in neutrophils determined by western blotting analysis. Representative western blotting photographs of the normal-control, sepsis-control, and sepsis + XBJ groups for the indicated time points were shown above. The equal loading of proteins was illustrated by the *β*-actin bands and bar graphs were obtained by densitometry of annexin A1/actin band densities. Data represented the mean ± SD of 6 rats examined for each group. *P < 0.05, compared with the sepsis-control group at the same time point; ^#^ *P* < 0.05, compared with the sepsis-control group at 6 hours after injection; and ^★^ *P* < 0.05, compared with the sepsis + XBJ group at 6 or 12 hours after injection.

To explore the effect of XBJ on cytokine-mediated inflammatory response to *A. baumannii* sepsis, the expression of anti-inflammatory factor annexin A1 in neutrophils was first determined by western blotting. As shown in Figure 4, annexin A1 protein level was significantly increased in sepsis-control group as compared with normal-control group for the same time period (*P* < 0.05). The stimulation was consistent with the length of injection time, as the increase in annexin A1 expression was shown time-dependently from 6 hrs to 24 hrs after inoculation (*P* < 0.05). Treatment of XBJ added a positive effect on the elevation of annexin A1 expression when simultaneously administrated with *A. baumannii *(*P* < 0.05), and the enhancement amplitude was most pronounced in sepsis + XBJ group at 24 hrs after injection.

### 3.5. Immunohistochemical Staining of Annexin A1 in Lungs in the Process of Sepsis

**Figure 5:**
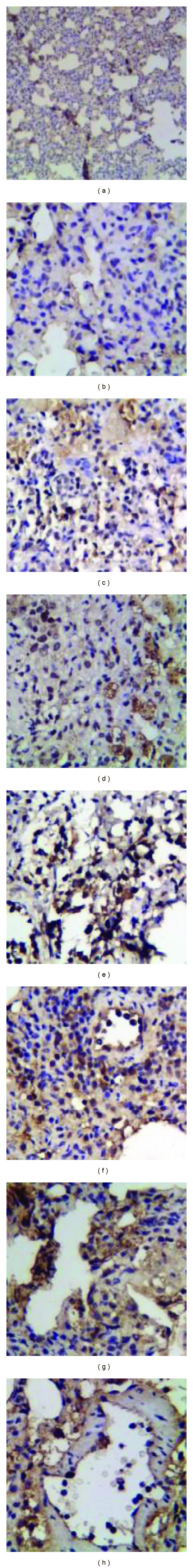
Representative sections showing immunohistochemical expression of annexin A1 in lung tissues from rats in the (a) and (b) normal-control, (c)–(e) sepsis-control, and (f)–(h) sepsis + XBJ groups at 6 (c) and (f), 12 (d) and (g), and 24 (e) and (h) hours after injection, respectively. annexin A1 staining was very limited in control lung, and an improved immunoreactivity could be seen, mainly localized to the cytoplasm of infiltrated neutrophils and hyperplastic alveolar epithelial cells where exudate and fibroplastic proliferation were obviously observed in sepsis-control groups. Notable observation was a more intensely positive reaction for annexin A1 in sepsis + XBJ groups than that of sepsis-control groups, although the number of inflammatory cells was significantly reduced by XBJ administration. Original magnification: (a) ×100 and (b)–(h) ×400.

To address the question of whether in the tissues increased annexin A1 protein expression could be observed, immunohistochemical staining of lungs was conducted. We found that a very low annexin A1 staining occurred in lung tissues from normal-control group, and positive reactivity only restricted to cytoplasm of some alveolar epithelial cells (Figures 5(a) and 5(b)). For lungs harvested from sepsis-control groups, annexin A1 concentration was elevated and the positive spots distributed unevenly in lung tissue as the inoculation time prolonged from 6 hrs to 24 hrs (Figures 5(c), 5(d), and 5(e)). In particular, alveolar epithelial cells of the lungs in the sepsis + XBJ groups showed more intensely positive reaction for annexin A1 than those of the sepsis-control groups at the same time points (Figures 5(f), 5(g), and 5(h)). Although the number of inflammatory cells was reduced by XBJ administration, strong annexin A1 reactivity was observed in some areas, where occasionally infiltrating cells occurred (Figures 5(f), 5(g), and 5(h)). These results were highly parallel to those of western blotting, and a quantitative analysis of annexin A1-positive spots in lung tissues was represented in Table 1. Overall, data showed that in rats with *A. baumannii* infection, annexin A1 was upregulated in a time-dependent fashion during the course of sepsis and XBJ added a positive effect on this anti-inflammatory enhancement activity by an early administration.

### 3.6. Plasma Levels of IL-8 and TNF-*α* in the Process of Sepsis and Their Modulation by XBJ

**Figure 6:**
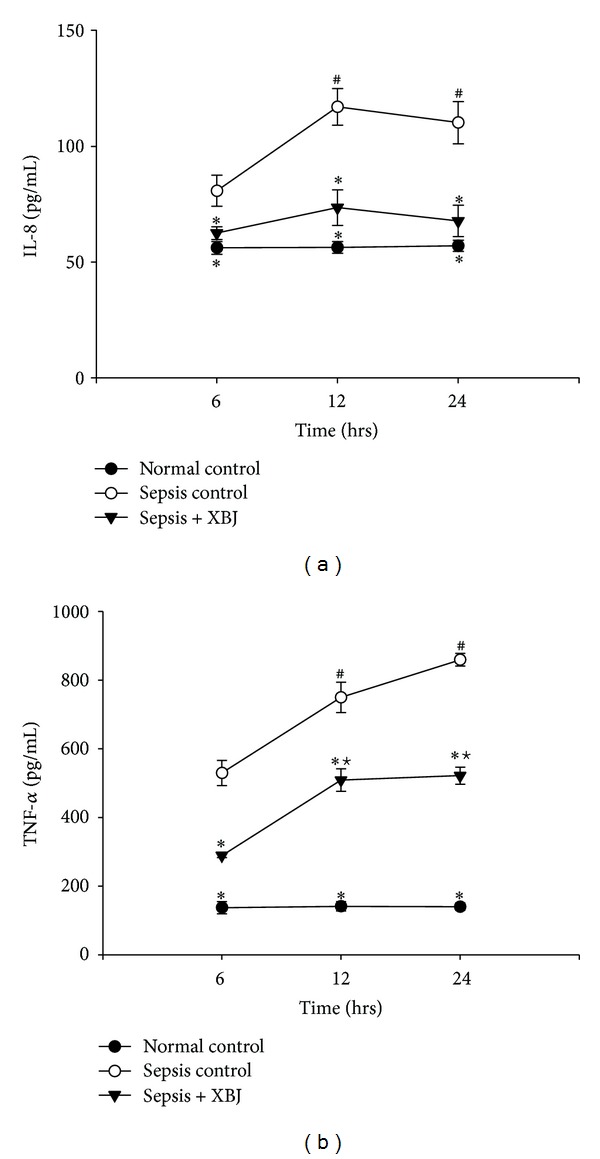
Enzyme-linked immunosorbent assay (ELISA) for serum levels of (a) IL-8 and (b) TNF-*α*. Expression levels of IL-8 and TNF-*α* in the normal-control, sepsis-control, and sepsis + XBJ groups at 6, 12, and 24 hours after injection were shown. Results indicated elevations of IL-8 and TNF-*α* in plasmas of rats of sepsis-control groups compared with those of normal-control groups at each monitored time point. The elevations of the two proinflammatory cytokines were both reduced after the administration of XBJ and they were most prominent in the sepsis + XBJ group at 24 hours after injection. *P < 0.05, compared with the sepsis-control group at the same time point; ^#^ *P* < 0.05, compared with the sepsis-control group at 6 hours after injection; and ^★^ *P* < 0.05, compared with the sepsis + XBJ group at 6 hours after injection.

We finally examined the effect of XBJ on proinflammatory cytokine productions in the process of *A. baumannii* sepsis. As shown in Figure 6, IL-8 and TNF-*α* cytokines levels were relatively low in control rats; however, both cytokines were increased in the sepsis-control groups, with the enhancement being most significant by 12 hrs for IL-8 and 24 hrs for TNF-*α* after inoculation (Figures 6(a) and 6(b)). Treatment with XBJ led to a significant reduction in both cytokines releases, as compared with the sepsis-control group at the same time point, with the inhibition rate of IL-8 by 22.7%, 37.2%, and 38.5% and TNF-**α** by 45.4%, 32.1%, and 39.3%, at 6, 12, and 24 hrs after injection, respectively. Thus, it is both perfect for “short” and “long” administrations of XBJ to prevent proinflammatory cytokine productions in the progress of sepsis induced by *A. baumannii*.

## 4. Discussion

XBJ is an intravenous injection consisting of five TCMs selected out from thirty-six traditional Chinese herb compound formulas. It has been extensively used for treating sepsis or MODS in China based on the theory of “antibacteria, antiendotoxin, and anti-inflammatory simultaneously.” Numerous studies have investigated the pharmacological effects of XBJ [14–17]; however, whether it could exert preventing effect on the development of *A. baumannii* infection otherwise progressing to sepsis or severe sepsis is currently poorly understood. Our data from the present study showed that rats with XBJ and *A. baumannii* concurrently administration elicited improved abnormal symptoms or signs and an early drop of the bacterial load in the peritoneal cavity, compared to those of rats with *A. baumannii* infection alone. Histopathological study further confirmed that XBJ treatment persistently attenuated lung histopathological changes, alveolar hemorrhage, and inflammatory cells infiltration. It was evidenced, therefore, that XBJ interference by early prophylactic administration was helpful and even necessary in minimizing the *A. baumannii* exposure.

In exploring the mechanisms by which XBJ protected rats from sepsis challenged with *A. baumannii*, the dynamic modulations of both anti-inflammatory and proinflammatory cytokines/mediators levels were investigated. Since IL-8 and TNF-*α* were demonstrated to be necessary to initiate an effective inflammatory process and could be evaluated as good parameters to predict the outcome of sepsis [27, 28], plasma levels of these two proinflammatory cytokines of rats were thus measured in the present study. Referring to anti-inflammatory mediators, increasing interest has been shown for annexin A1 due to its endogenous proresolving properties. This glucocorticoid-regulated protein annexin A1, mainly expressing in subcellular granules of neutrophils and monocytes [29], has been implicated in a number of biological events, including both acute [30] and chronic [31] inflammations, leukocyte trafficking [32], monocyte migration [33], and apoptotic leukocytes clearance [34]. annexin A1 may also affect a number of mediators that are involved in the inflammatory response, including IL-10, cyclooxygenase-2 (Cox-2), and inducible nitric oxide synthase (iNOS) [35, 36]. Nevertheless, the response of annexin A1 in the process of *A. baumannii *infection and whether this novel mediator is able to be used as a new biomarker in the diagnosis of sepsis remain to be elucidated. Therefore, we measured not only the dynamic changes of its protein level in neutrophils but also the expression in cytoplasmic movements of lung tissue, which is the most often affected organ in MODS after sepsis [37], during the course of sepsis and after therapy.

We demonstrated that the expression of annexin A1, as well as the plasma levels of proinflammatory cytokines, IL-8, and TNF-*α*, was elevated in septic rats compared to normal rats, revealing that both types of cytokine play a role from the very beginning of this life-threatening condition. These results were consistent with prior studies, by showing that secretions of both pro- and anti-inflammatory mediators occur as a simultaneous immune response program initiated early in the course of the disease either in sepsis patients [7, 8] or animals [38, 39]. However, our results differed from previous studies in which it had been shown that annexin A1 decreased in sepsis patients [40] or obese patients with chronic inflammatory phenotype [41]. Considering the inconsistencies between these studies, one possible explanation involves the fact that these early studies predominantly focused on whole animal or patient approaches; they did not determine the level of annexin A1 directly in neutrophils or lung tissue involved in development of sepsis as described in our present study. Another potential interpretation is related to the biphasic model pathogenesis of the most severe form of sepsis, that is, an initial proinflammatory phase followed by an anti-inflammatory response [42, 43]. In the present study, we investigated the level of annexin A1 during the first 24 hrs following *A. baumannii* infection, and cytokines determined from prior study were in patient hospitalized for neonatal sepsis. We hypothesized that the role anti-inflammatory cytokines playing is a highly dynamic biological process and can be differently modulated in the different phases as the sepsis progresses.

Here, an interesting and noticeable finding must be rendered is the ability of XBJ to increase annexin A1 expression at each monitored time point determined by both western blotting and immunohistochemical analysis, when compared with that of sepsis-control group. Considering that annexin A1 plays a critical role in a number of immune-related cellular processes, thus it is reasonable to assume that annexin A1 modulation is responsible for at least one mechanism related to the anti-inflammatory effect of XBJ. To the best of our knowledge, we are the first to demonstrate that the level of annexin A1 is elevated during *A. baumannii* sepsis progression in a rat model and that a novel activity of XBJ to modulate annexin A1 expression, which adds a new component to its mechanistic frame. However, as for the molecular mechanism and concrete signal transduction process in XBJ regulating network, further studies are still needed.

The continuous enhancement of anti-inflammatory cytokine production during the first 24 hrs following injection suggested an inflammation compromised state was likely associated with these infected rats by XBJ early interference. This was further confirmed by ELISA results to show that IL-8 and TNF-*α* were suppressed to a relative low level by XBJ treatment at various time points, which were consistent to the anti-inflammatory effect of XBJ shown by previous studies. For example, Wang et al. found that XBJ could effectively inhibit high-mobility group box-1 protein (HMGB1) synthesis and release in renal tissues and prevent the development of acute kidney injury induced by serious scald injury [44]. Qi et al. had shown that treatment with XBJ decreased the secretion of TNF-*α*, IL-6, and IL-8, thereby showing a protective effect in patients with severe pneumonia [45]. XBJ was also found to inhibit IL-6 and TNF-*α* secretion in mice with lipopolysaccharide-induced acute lung injury (ALI) [15] and restore the acquired immune suppression due to overactive proinflammatory cytokine productions in patients with MODS [46]. In the present study, we investigated the suppressive effect of XBJ on the development of *A. baumannii*-induced sepsis in rats by not only increasing the expression of annexin A1 but also decreasing the serum release of either IL-8 or TNF-*α*. This is critically important, since persistently high or increasing levels of proinflammatory cytokines have been associated with multiple organ-system dysfunctions [6, 47] and an early and sustained deficient in immunosuppression has been found to predict mortality of sepsis [48]. Thus, we considered that once with XBJ treatment from the first moments of infection, the uncontrolled release of endogenic proinflammatory mediators was suppressed. As a result, the vacious cycle of inflammation onset was interrupted and the development of SIRS was blocked, and meanwhile, the detectable symptoms were eventually relieved.

To sum up, the present study proved that prophylactic administration of XBJ can effectively alleviate the symptoms and tissue injury in a rat model of *A. baumannii* infection, otherwise might develop to sepsis or severe sepsis. This preventive effect of XBJ in the progress of sepsis may be related to the modulation of cytokine-mediated inflammatory response. The successful interference by early administration of XBJ gives new insight into the clinical use of XBJ and also highlights the need for further studies to clarify its therapeutic effect after the onset of *A. baumannii* sepsis.

## Figures and Tables

**Table 1:** Pathological scores of lung annexin A1 staining from different groups and for different time periods.

Groups	Treatment time periods (hrs)		
	6	12	24
Normal control	1.25 ± 0.68*	1.18 ± 0.75*	1.31 ± 0.70*
Sepsis control	4.50 ± 1.26	4.75 ± 1.18	5.25 ± 1.73
Sepsis + XBJ	8.06 ± 1.56*	8.50 ± 1.75*	8.37 ± 1.85*

Tissue sections were stained with the anti-annexin A1 antibody and the evaluation of pathological scores was determined by two pathologists. Data represented the mean ± SD of 6 rats examined for each group. *P < 0.05, compared with the sepsis-control group.
